# Pulsed field or cryoballoon ablation for paroxysmal atrial fibrillation—insights from acute and chronic electroanatomic remapping in the randomized SINGLE-SHOT CHAMPION trial

**DOI:** 10.1093/europace/euag167

**Published:** 2026-07-03

**Authors:** Thomas Kueffer, Sven Knecht, Elias Ayadi, David Spreen, Salik ur Rehman Iqbal, Gregor Thalmann, Patrick Badertscher, Jens Maurhofer, Philipp Krisai, Nikola Kozhuharov, Peter Jüni, Corinne Jufer, Helge Servatius, Hildegard Tanner, Michael Kühne, Laurent Roten, Tobias Reichlin, Christian Sticherling

**Affiliations:** Department of Cardiology, Inselspital, Bern University Hospital, University of Bern, Bern, Switzerland; SITEM Center for Translational Medicine and Biomedical Entrepreneurship, University of Bern, Bern, Switzerland; Department of Cardiology, University Hospital Basel, University Basel, Basel CH-4031, Switzerland; Department of Cardiology, University Hospital Basel, University Basel, Basel CH-4031, Switzerland; Department of Cardiology, University Hospital Basel, University Basel, Basel CH-4031, Switzerland; Department of Cardiology, Inselspital, Bern University Hospital, University of Bern, Bern, Switzerland; Department of Cardiology, Inselspital, Bern University Hospital, University of Bern, Bern, Switzerland; Department of Cardiology, University Hospital Basel, University Basel, Basel CH-4031, Switzerland; Department of Cardiology, Inselspital, Bern University Hospital, University of Bern, Bern, Switzerland; Department of Cardiology, University Hospital Basel, University Basel, Basel CH-4031, Switzerland; Department of Cardiology, Inselspital, Bern University Hospital, University of Bern, Bern, Switzerland; Clinical Trial Service Unit and Epidemiological Studies Unit, Nuffield Department of Population Health, University of Oxford, Oxford, UK; Department of Cardiology, Inselspital, Bern University Hospital, University of Bern, Bern, Switzerland; Department of Cardiology, Inselspital, Bern University Hospital, University of Bern, Bern, Switzerland; Department of Cardiology, Inselspital, Bern University Hospital, University of Bern, Bern, Switzerland; Department of Cardiology, University Hospital Basel, University Basel, Basel CH-4031, Switzerland; Department of Cardiology, Inselspital, Bern University Hospital, University of Bern, Bern, Switzerland; Department of Cardiology, Inselspital, Bern University Hospital, University of Bern, Bern, Switzerland; Department of Cardiology, University Hospital Basel, University Basel, Basel CH-4031, Switzerland

**Keywords:** Paroxysmal atrial fibrillation, Pulsed-field ablation, Cryoballoon ablation, 3D mapping, Lesion extent, Lesion durability

## Abstract

**Aims:**

The SINGLE SHOT CHAMPION multicentre trial compared the effectiveness of pulsed-field ablation (PFA) and cryoballoon ablation (CBA) for pulmonary-vein isolation (PVI) in patients with paroxysmal atrial fibrillation (AF). In a prespecified substudy, post-ablation three-dimensional electroanatomic mapping (3D-EAM) was performed, and repeat-procedure mapping data were analysed.

**Methods and results:**

Patients were randomized 1:1 to fluoroscopy-guided PVI with PFA or CBA. The first 25 patients in each group underwent high-density 3D-EAM immediately after ablation. Acute PV isolation and lesion geometry were assessed using bipolar voltage thresholds of 0.1, 0.2, and 0.5 mV. Lesion durability was evaluated at clinically indicated repeat ablation. In acute post-ablation mapping, residual PV conduction was seen in 4/25 (16%) CBA patients and 0/25 PFA patients (*P* = 0.11). Inadvertent posterior wall conduction block occurred after PFA in 3 (12%) patients with small atria (mean LA volume 30.7 mL). PFA created larger left-sided lesions (antral area <0.5 mV: 5.7 mm^2^ [IQR 4.6–6.5] vs. 4.4 mm^2^ [IQR 2.5–5.5], *P* = 0.026) and narrower posterior wall channels (14.0 mm [IQR 10.5–20.6] vs. 24.0 mm [IQR 19.1–26.9], *P* < 0.001), indicating wide-antral isolation. At redo (*n* = 48), durable PVI was seen in 4/26 (15%) PFA and 3/22 (14%) CBA patients and in 63/102 (62%) veins after PFA and 52/82 (63%) after CBA (*P* = 0.88).

**Conclusion:**

Fluoroscopy- and electrocardiogram-guided PVI can result in incomplete PV isolation after CBA or unintentional posterior wall block after PFA. In the acute mapping cohort, PFA yielded broader left-sided lesions than CBA; in a separate redo cohort, chronic PV durability was similar for both modalities.

## Graphical Abstract

**Graphical Abstract:**
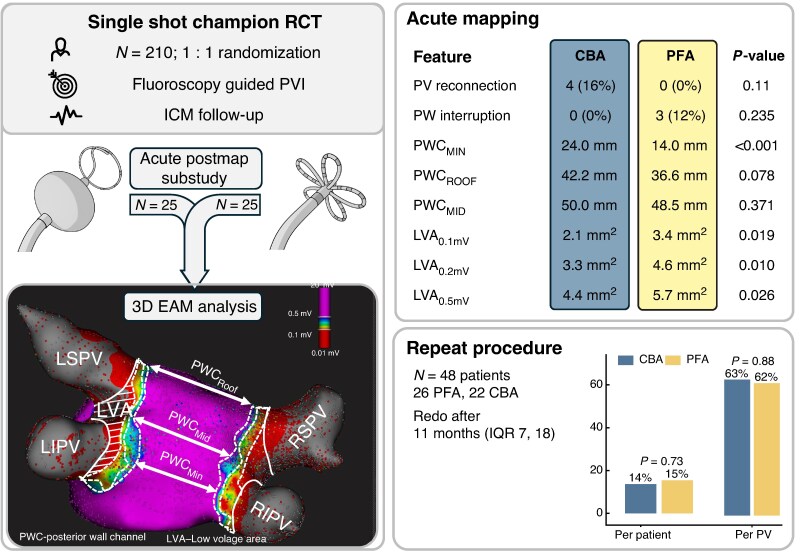

What's new?

- In the randomized SINGLE SHOT CHAMPION trial, post-ablation 3D mapping showed pulsed-field ablation (PFA) created broader antral left-sided lesions and a narrower posterior wall channel than cryoballoon ablation (CBA), with inadvertent posterior wall block in 12% of PFA patients with small atria.
- Despite high operator experience, fluoroscopy-guided CBA left residual pulmonary-vein conduction in 16% of patients.
- At clinically indicated repeat procedures, per-vein durability was nearly identical (62% PFA vs. 63% CBA); larger left-atrial volume independently predicted reconnection.

## Introduction

Recent randomized trials have demonstrated that pulsed-field ablation (PFA) achieves pulmonary-vein isolation (PVI) with efficacy comparable to established thermal technologies, while offering a favourable safety profile.^1–3^ The underlying lesion characteristics and spatial distribution created by single-shot ablation catheters remain incompletely characterized.

In current practice, both cryoballoon and PFA procedures are primarily guided by fluoroscopy, and pacing and signal-based endpoint verification, with limited use of 3D mapping. As a result, the precise extent of ablation, completeness of PV isolation, or unintended ablation of adjacent atrial tissue cannot be directly visualized. Incomplete PVI or unintended posterior wall interruption may therefore go unnoticed despite apparent procedural success.^4–6^

The SINGLE SHOT CHAMPION trial directly compared CBA and PFA for first-time PVI in patients with paroxysmal atrial fibrillation under continuous rhythm monitoring during follow-up via an implantable cardiac monitor (ICM). In a substudy, high-density 3D mapping was performed immediately after ablation in a subset of patients, providing detailed insights into acute lesion extent. Mapping data from clinically indicated repeat procedures further allowed assessment of pulmonary-vein durability and reconnection patterns.

The primary objective of this study was to compare the extent of acute antral lesion size around the PVs for the two modalities.

## Methods

### Trial design and oversight

The SINGLE SHOT CHAMPION (SSC, ClinicalTrials.gov number, NCT05534581) trial was a randomized, investigator-initiated comparison of pulsed-field ablation (PFA) with cryoballoon ablation (CBA) for fluoroscopy-guided pulmonary-vein isolation (PVI) in paroxysmal atrial fibrillation (AF) patients.^2^ The present work is a prespecified electroanatomical mapping (EAM) substudy after ablation during the index procedure and a post-hoc analysis of findings at clinically indicated repeat procedures of the entire cohort. The study protocol was approved by the local ethics committees, adhered to the Declaration of Helsinki, and was embedded in the main SSC statistical analysis plan. All operators had >6 months experience with PFA and >2 years’ experience with CBA before enrolment, respectively.

### Patients

The first 25 patients in each group underwent adjunctive high-density left-atrial 3D-EAM (Rhythmia, Boston Scientific) immediately after completion of PVI during the index procedure. To prevent confounding, no additional energy deliveries were allowed after left-atrial mapping, even when reconnection was detected on 3D-EAM.

For the repeat-procedure analysis, SSC participants who underwent a left-atrial redo ablation for symptomatic arrhythmia recurrence until December 2025 were included. Bipolar voltage-guided lesion durability assessment was performed.

### Index ablation procedure

PFA was delivered with the pentaspline 31-mm or 35-mm FARAWAVE catheter via a 13-F steerable sheath; each application consisted of pulse trains (2.0 kV) delivered unsynchronized to the cardiac cycle. CBA was performed with the Arctic Front 23- or 28-mm balloon via a 12-F sheath using a ‘time-to-effect + 2 min’ strategy.^7^ Acute PVI was verified by the absence of local electrograms within each pulmonary-vein ostia and testing of exit block using 10 V at 2 ms pacing.^8^ No left-atrial ablation outside the pulmonary veins was performed. Details of periprocedural patient management, *trans*-septal access, and anticoagulation have been reported previously.^2^

### Quantitative 3D electroanatomical lesion assessment

Immediately after confirmation of acute PVI with the treatment catheter, a 3D-EAM using an ultra-high-density mapping catheter in combination with a 3D mapping system (Orion™ and Rhythmia™, Boston Scientific) was performed. Mapping was performed in paced atrial rhythm or sinus rhythm.

High-density maps were post-processed by a blinded core laboratory using a predefined workflow. Areas with sub-threshold bipolar voltage values extending from the PV ostium into the atrium were measured. PV ostia were defined at the visual intersection of the PVs with the left atrium.^9^ Three different thresholds were used for area measurement: <0.1, <0.2, and <0.5 mV. Border-zone area was calculated by subtracting the smaller dense-scar area A_0.1_ from the larger A_0.5_ area. Anterior areas were disregarded due to far-field signals and ridge mapping artefacts.

**Figure 1:**
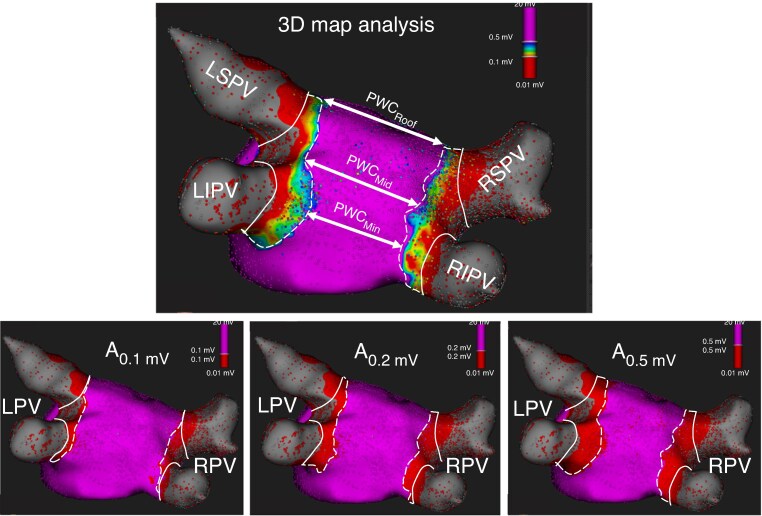
3D map analysis. Posterior wall cannels were measured at the roof and mid-posterior wall in addition to the isthmus. Lesion extent and border zone were characterized using three different cut-off values (0.1, 0.2, and 0.5 mV).

The residual posterior wall channel—the distance between the inner red borders of the left- and right-sided PVI lesion sets—was measured at the roof and posterior, and at the minimal distance, using a bipolar voltage cut off of 0.1 mV (***Figure 1***). To evaluate inter-observer reliability, 10 randomly selected maps were analysed by a second observer, and intraclass correlation coefficients were calculated for both PW channel and border-zone metrics.

### Repeat-procedure mapping

At clinically indicated repeat ablation of the entire cohort, high-density left-atrial maps were obtained with commercially available multielectrode catheters before ablation. System selection was based on operator preference. Pulmonary-vein reconnection was assessed on a per-vein and per-patient basis. Each PV circumference was divided into four quadrants to localize reconnection sites. Mapping system heterogeneity prevents us from accurately determine lesion regression.

### Statistical analysis

Continuous variables are presented as mean ± standard deviation (SD) or median with interquartile range (IQR) and were compared using Student’s *t*-test or Wilcoxon rank-sum test, as appropriate. Categorical variables were compared using the χ^2^ test or Fisher’s exact test. For the per-vein analyses, mixed-effects logistic regression models were used to account for clustering within patients (random intercept per patient). Two-sided *P* values < 0.05 were considered statistically significant. Statistical analyses were performed using R version 4.5.0 (R Foundation for Statistical Computing, Vienna, Austria).

## Results

### Study population

Fifty consecutive patients (25 PFA, 25 CBA) underwent acute 3D-EAM and 48 patients underwent a left-atrial repeat ablation; baseline clinical characteristics were balanced between the mapping- and the non-mapping and the redo- and non-redo group (***Table 1***). The PFA group received a median of 40 applications (IQR 34–44), whereas the CBA group received a median of 6 freezes (IQR 5–7) and a total freezing time of 18.5 min (IQR 15.8–22.0).

**Table 1:** Baseline patient characteristics

Variable	Mapping	No mapping	*P*	Redo	No redo	*P*
*n*	50	160		48	162	
Randomized to PFA	25 (50.0)	80 (50.0)	1.000	26 (54.2)	79 (48.8)	0.622
Age—year	62.5 [55.2, 69.5]	65.0 [58.8, 70.0]	0.149	65.0 [57.0, 70.2]	65.0 [58.0, 70.0]	0.918
Male sex	40 (80.0)	111 (69.4)	0.155	37 (77.1)	114 (70.4)	0.465
Body-mass index—kg/m^2^	27.8 [24.7, 30.1]	26.4 [23.5, 29.4]	0.113	26.5 [24.0, 28.7]	26.6 [23.8, 29.8]	0.641
CHA2DS2-VASc score	1.0 [1.0, 3.0]	2.0 [1.0, 3.0]	0.265	2.0 [1.0, 3.0]	2.0 [1.0, 3.0]	0.938
Currently smoker	5 (10.0)	17 (10.6)	1.000	6 (12.5)	16 (9.9)	0.597
Hypertension	29 (58.0)	85 (53.1)	0.626	25 (52.1)	89 (54.9)	0.744
Vascular disease	7 (14.0)	21 (13.1)	0.816	6 (12.5)	22 (13.6)	1.000
History of congestive heart failure	3 (6.0)	9 (5.6)	1.000	3 (6.2)	9 (5.6)	1.000
Diabetes	6 (12.0)	16 (10.0)	0.791	4 (8.3)	18 (11.1)	0.789
Previous stroke, TIA, or peripheral embolism	1 (2.0)	10 (6.2)	0.466	4 (8.3)	7 (4.3)	0.278
Obstructive sleep apnea syndrome	6 (12.0)	22 (13.8)	1.000	8 (16.7)	20 (12.3)	0.470
No. of years since first diagnosis of atrial fibrillation	0.8 [0.2, 2.5]	1.2 [0.4, 4.0]	0.080	1.3 [0.3, 4.3]	1.0 [0.4, 3.5]	0.765
Antiarrhythmic drug	7 (14.0)	39 (24.4)	0.169	13 (27.1)	33 (20.4)	0.326
Amiodarone	4 (8.0)	14 (8.8)	1.000	3 (6.2)	15 (9.3)	0.769
Class IC antiarrhythmic drugs	3 (6.0)	20 (12.5)	0.299	9 (18.8)	14 (8.6)	0.064
Sotalol	0 (0.0)	6 (3.8)	0.339	1 (2.1)	5 (3.1)	1.000
Anticoagulant use	45 (90.0)	144 (90.0)	1.000	41 (85.4)	148 (91.4)	0.272
Direct oral anticoagulant	43 (86.0)	140 (87.5)	0.810	40 (83.3)	143 (88.3)	0.461
Vitamin K antagonist	2 (4.0)	4 (2.5)	0.630	1 (2.1)	5 (3.1)	1.000
Left ventricular ejection fraction—%	60.0 [57.8, 62.8]	60.0 [57.5, 64.0]	0.523	60.0 [55.8, 63.5]	60.0 [58.0, 64.0]	0.961
Left-atrial volume index—mL/m^2^	35.4 [28.0, 41.0]	34.0 [28.0, 40.3]	0.598	36.2 [27.5, 45.8]	34.0 [28.0, 39.0]	0.195
Left-atrial volume—mL	70.6 [58.6, 88.2]	66.2 [55.2, 80.3]	0.483	72.8 [54.7, 90.8]	65.4 [56.7, 79.9]	0.212

### Acute post-ablation mapping

**Figure 2:**
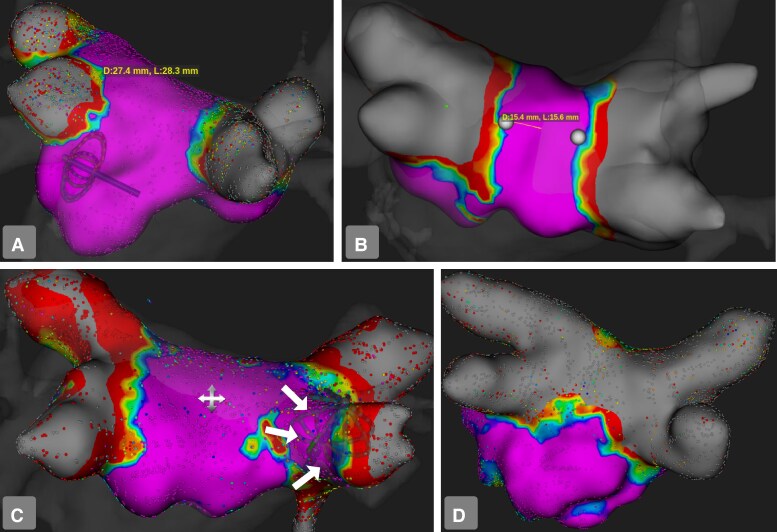
Acute post-ablation mapping examples. Ostial PVI (panel A) after Cryoballoon ablation and wide-antral PVI (panel B) after pulsed-field ablation. Residual PV connection (panel C) after fluoroscopy-guided Cryoballoon PVI. Inadvertent posterior wall interruption (panel D) after pulsed-field ablation. PVI = Pulmonary-vein isolation.

Residual PV conduction was documented in four veins (four patients, 16%) after CBA (two right-inferior, one left-inferior, one left-superior) and in none after PFA (*P* = 0.11). The minimal posterior wall lesion distance was significantly narrower after PFA than after CBA (24.0 mm, IQR 19.1–26.9, vs. 14.0 mm, IQR 10.5–20.6, *P* < 0.001), indicating more antral PVI (***Figure 2***). Roof lesion border distances followed this observation (***Table 2***). Ablated areas around the left pulmonary veins were larger after PFA (5.7 mm^2^, IQR 4.6–6.5 vs. 4.4 mm^2^, IQR 2.5–5.5, *P* = 0.026), and similar around the right-sided veins for both modalities (5.1 mm^2^ IQR 3.8–6.0 for PFA vs. 4.2 mm^2^, IQR 2.5–6.0 for CBA, *P* = 0.419). The border-zone area, defined as the area with electrograms between 0.1 and 0.5 mV, was similar in both groups. Inadvertent posterior wall conduction block occurred in 3/25 PFA patients (12%) and 0/25 CBA patients (*P* = 0.23) and was associated with smaller left-atrial volume (mean LA volume 30.7 mL vs. 74.8 mL; *P* = 0.001). The lowest intraclass correlation coefficient was 0.92.

**Table 2:** Procedural data and lesion extent

Variable	CBA	PFA	*P*
*n*	25	25	
General anaesthesia	0 (0.0)	1 (4.0)	1.000
31 mm PFA catheter	0 (0.0)	25 (100.0)	<0.001
28 mm CBA catheter	25 (100.0)	0 (0.0)	<0.001
Radiofrequency CTI Ablation	3 (12.0)	4 (16.0)	1.000
Procedure duration—min	83.0 [63.0, 104.0]	69.0 [57.0, 85.0]	0.070
Transpired ablation time—min	35.0 [25.0, 49.0]	23.0 [20.0, 32.0]	0.001
Transpired mapping time—min	12.0 [8.0, 17.0]	13.0 [9.0, 20.0]	0.655
Fluoroscopy time—min	15.0 [11.2, 16.9]	15.5 [12.6, 21.3]	0.393
Fluoroscopy dose—cGy*cm^2^	533.0 [315.0, 867.0]	417.0 [288.0, 610.0]	0.207
Acute PVI as per 3D-EAM	21 (84.0)	25 (100.0)	0.110
Unintentional posterior wall interruption	0 (0.0)	3 (12.0)	0.235
Posterior wall channel			
Posterior wall channel width, minimal—mm	24.0 [19.1, 26.9]	14.0 [10.5, 20.6]	0.001
Posterior wall channel width, roof—mm	42.2 [31.6, 49.4]	36.6 [32.7, 39.3]	0.078
Posterior wall channel width, mid-PW—mm	50.0 [43.6, 52.9]	48.5 [41.9, 51.1]	0.371
Lesion size			
LPV <0.1 mV area—mm^2^	2.1 [0.9, 3.5]	3.4 [2.2, 4.5]	0.019
LPV <0.2 mV area—mm^2^	3.3 [1.7, 4.3]	4.6 [3.3, 5.6]	0.010
LPV <0.5 mV area—mm^2^	4.4 [2.5, 5.5]	5.7 [4.6, 6.5]	0.026
RPV <0.1 mV area—mm^2^	1.8 [0.5, 3.4]	2.8 [1.9, 3.5]	0.230
RPV <0.2 mV area—mm^2^	3.2 [1.3, 4.4]	3.8 [2.9, 4.4]	0.306
RPV <0.5 mV area—mm^2^	4.2 [2.5, 6.0]	5.1 [3.8, 6.0]	0.419
LPV border-zone area—mm^2a^	1.8 [1.3, 2.4]	2.3 [1.6, 2.9]	0.136
RPV border-zone area—mm^2a^	2.1 [1.8, 2.6]	1.9 [1.6, 2.6]	0.604

Numbers are no. (%) or median [IQR] as appropriate. IQR = interquartile range

^a^Defined as the area with a recorded voltage of >0.1 and <0.5 mV

### Repeat procedures

The redo cohort is distinct from the acute mapping cohort and reflects all SSC participants who underwent a clinically indicated repeat procedure, regardless of whether they had been part of the index-procedure mapping substudy.

A total of 48 SSC participants (26 PFA, 22 CBA) underwent clinically indicated redo ablation a median of 10.8 months (IQR 7.1, 17.9) after the index procedure.

Atrial fibrillation was the most common arrhythmia leading to a repeat procedure (77%, ***Table 3***). Roof-dependent and peri-mitral flutter were observed in two and one case(s), respectively. No roof-dependent flutter was observed in the three patients with unintended posterior wall interruption; one showed posterior wall reconnection on repeat procedure.

**Table 3:** Repeat procedures

Variable	Overall	CBA	PFA	*P*
*n*	48	22	26	
Time to redo—months	10.8 [7.1, 17.9]	12.4 [7.4, 21.1]	8.3 [6.9, 17.0]	0.234
Arrhythmia leading to redo				
AF leading to redo	37 (77.1)	17 (77.3)	20 (76.9)	1.000
Typical flutter leading to redo	3 (6.2)	1 (4.5)	2 (7.7)	1.000
Atypical flutter leading to redo	3 (6.2)	2 (9.1)	1 (3.8)	0.587
Atrial tachycardia leading to redo	5 (10.4)	2 (9.1)	3 (11.5)	1.000
Atypical flutter mechanisms				
Roof-dependent flutter	1 (2.1)	1 (4.5)	0 (0.0)	0.458
Peri-mitral flutter	2 (4.2)	2 (9.1)	0 (0.0)	0.205
Durable PVI	7 (14.6)	3 (13.6)	4 (15.4)	1.000
Repeat ablation lesion set				
CTI ablation	13 (27.1)	9 (40.9)	4 (15.4)	0.059
Posterior wall ablation	21 (43.8)	8 (36.4)	13 (50.0)	0.393
Posterior mitral isthmus line	1 (2.1)	0 (0.0)	1 (3.8)	1.000
Anterior mitral isthmus line	3 (6.2)	3 (13.6)	0 (0.0)	0.089
Superior vena cava isolation	3 (6.2)	1 (4.5)	2 (7.7)	1.000

Numbers are no. (%) or median [IQR] as appropriate. IQR = interquartile range

**Figure 3:**
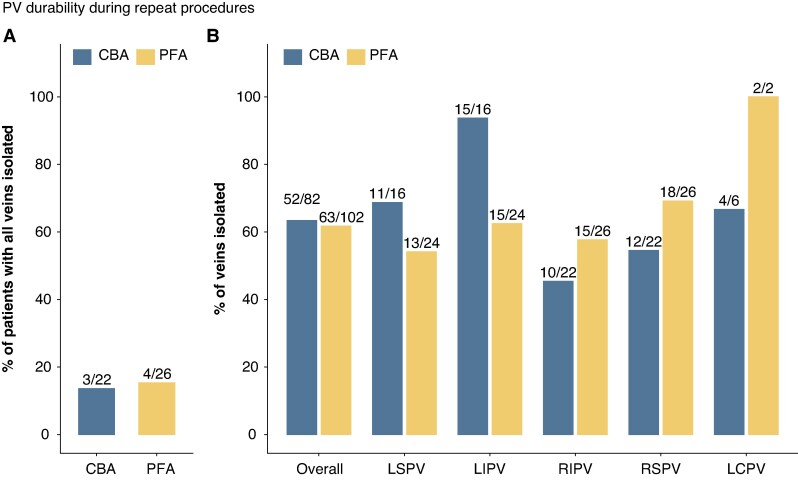
PV durability during repeat procedures. Per-patient (panel A) and per-PV (panel B) lesion durability recorded during clinically indicated repeat procedures. Complete PVI was present in 13.6% after CBA and in 15.4% after PFA. PV durability was 63.4% for CBA and 61.8% for PFA.

**Figure 4:**
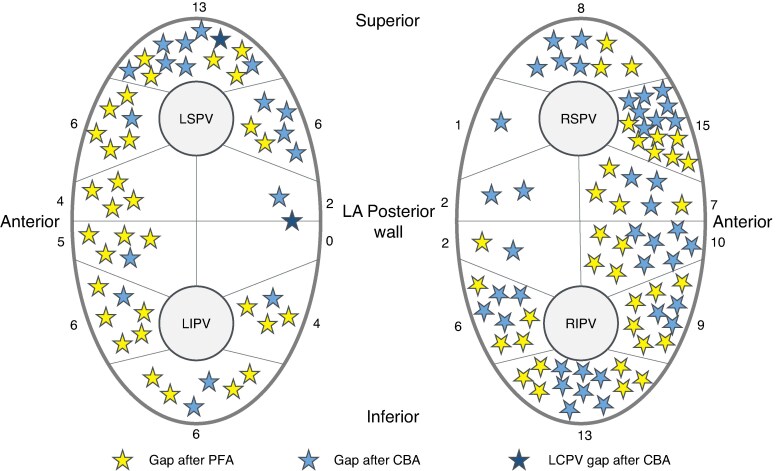
Reconnection patterns. PV reconnections were concentrated on the anterior, superior, and inferior aspects, regardless of technology. Especially, the anterior RSPV (*n* = 15), inferior RIPV (*n* = 13) and superior LSPV (*n* = 13) were common locations for reconnections. CBA = Cryoballoon ablation; LCPV = Left common pulmonary vein; PFA = Pulsed-field ablation.

3D electroanatomic mapping was performed in all cases, using Octaray or Pentaray catheters (Carto, Biosense Webster) in 39 of 48 patients, Sphere-9 (Affera, Medtronic) in 4, Varipulse (Carto, Biosense Webster) in 3, and Faraview (Rhythmia, Boston Scientific) in two patients. Durable isolation was observed in 115/184 (63%) veins, in 63/102 (62%) after PFA and in 52/82 (63%, *P* = 0.88) after CBA, corresponding to complete durable PVI in 15.4% vs. 13.6% of patients, respectively (*P* = 0.73, ***Figure 3***). The most frequent reconnection sites were the anterior aspect of all four veins, the inferior quadrant of the right-inferior vein, and the superior aspect of the LSPV (***Figure 4***). In standard anatomy, CBA had a tendency to fewer reconnections than PFA in the left-sided PVs (LSPV plus LIPV, 81% durable vs. 58% durable, *P* = 0.051). There was no difference between PFA and CBA in common ostia durability, defined as ≥10 mm from the ridge to the first bifurcation, with durable isolation observed in 4/6 (67%) after CBA and 2/2 (100%) after PFA (*P* = 1.00).

### Predictors of PV reconnection

In the per-vein analysis of 184 veins from 48 patients, larger left-atrial volume was independently associated with pulmonary-vein reconnection (OR per mL increase: 1.02, 95% CI 1.00–1.03; *P* = 0.01). Randomization group, procedure duration and 3D mapping were not associated with PV reconnection. Higher ablation intensity, defined as the number of applications per vein for PFA and freeze duration for CBA, standardized within each ablation modality, showed a trend towards more reconnections (OR 1.42, 95% CI 1.00–2.04; *P* = 0.05, Supplementary material online, table  *S1*).

## Discussion

This substudy of the SINGLE SHOT CHAMPION trial provides detailed electroanatomic mapping insights after fluoroscopy-guided single-shot PVI—data that are typically unavailable in routine practice. The main findings are: (1) PFA leads to wider, more antral left-sided lesions and a narrower posterior wall channel compared with CBA; (2) unintentional posterior wall interruption occurred in small atria treated with PFA; (3) fluoroscopy-guided CBA left residual PV conduction in 16% of patients despite high operator experience; and (4) larger left atria were associated with PV reconnection at clinically indicated repeat procedures.

### Lesion morphology and anatomic extent

Post-ablation mapping demonstrated significantly broader antral coverage after PFA around the left PVs, and a narrower residual PW corridor, whereas the border-zone area between 0.1 and 0.5 mV was similar in both groups. Kiuchi *et al.* previously associated larger isolated areas with better long-term outcomes after PVI.^10^ Although our study did not assess clinical success as a function of lesion area, the wider antral isolation observed with PFA (LPV <0.5 mV area 5.7 vs. 4.4 mm^2^; *P* = 0.03) supports the hypothesis that larger ablation zones may contribute to improved long-term efficacy.^2^

Lesion geometry after cryoballoon ablation has been described before as being antral.^11,12^ Kawamura *et al.* reported that CBA occasionally creates ‘notched’ lesions at the carina, whereas the overall lesion extent with PFA was comparable to combined thermal techniques (CBA, RFA, laser).^13,14^ Our findings align with these observations, demonstrating more antral left-sided isolation after PFA when directly compared to CBA, but similar coverage around the right-sided PV.

Because PFA induces reversible electroporation, a wider transition zone might be expected as electric field intensity decreases with distance.^15^ This effect was not evident in our data, as border-zone width was similar between groups, however, temporal lesion dynamics were not investigated.

### Limitations of fluoroscopy-only guidance

Unintentional PW interruption after PFA, observed in 12% of PFA patients, reflects lesion overlap in smaller atria. Going unnoticed in fluoroscopy-guided PVI, even minor lesion regression might create an iatrogenic PW channel, facilitating roof-dependent macro-reentry. Previous reports have linked reconnection of intentionally isolated posterior walls to roof-dependent tachycardia in up to 75% of cases.^16^ However, whether PFA leads to a higher rate of atypical flutters than thermal ablation is a matter of an ongoing debate with conflicting evidence.^17,18^ In our cohort, no roof-dependent flutter occurred in the three patients with unintended posterior wall interruption; this is a reassuring preliminary observation, but the small number of affected patients precludes firm conclusions about the long-term proarrhythmic risk of inadvertent extra-PV lesions.

Conversely, despite preprocedural CT and extensive cryoballoon experience, 4 of 25 CBA patients (16%) had incomplete PV isolation, highlighting that fluoroscopic PV occlusion and post-ablation pacing does not always ensure complete electrical isolation. While incomplete occlusion of smaller veins might hinder PVI in CBA, the broader lesion footprint of PFA may render acute isolation more robust.

Adjunctive 3D mapping could help avoid both scenarios by clarifying catheter position and lesion geometry before energy delivery, though its influence on long-term clinical outcome remains to be determined. A recent EHRA survey reported wide variability in the use and implementation of 3D mapping during PFA across European centres, underscoring the lack of consensus on its role. ^19–21^

### PV durability and predictors

At redo procedures, durable PVI per patient was achieved in 15.4% after PFA and 15.8% after CBA, and per-vein durability was 62% and 63%, respectively—roughly matching values from prior large cohorts, including Pott *et al.* (64% for CBA) and Scherr *et al.* (70% for PFA).^22,23^ These findings suggest that despite distinct acute lesions, chronic durability does not differ materially between modalities. Procedural workflow refinements such as the OLIVE protocol, using additional overlapping PFA applications per vein, have been associated with higher lesion durability and may represent one approach to address the persistent reconnection rates observed here.^24^

Beyond procedural optimization, the independent association of larger left-atrial volume with reconnection, irrespective of energy source, aligns with previous evidence and the recent EHRA/HRS/APHRS/LAHRS consensus, in which LA volume is a defining feature of atrial cardiomyopathy staging, suggesting that substrate, rather than lesion morphology alone, limits durable single-shot PVI.^25–28^ Taken together, these observations indicate that lesion morphology and size alone does not determine chronic isolation success and that the positioning of PFA as a default strategy for all AF ablation candidates remains a matter of ongoing debate.^29^

### Limitations

This substudy included 25 patients per group for acute mapping and 48 patients undergoing clinically indicated redo, which may introduce selection bias. Acute lesion geometry and chronic durability were assessed in two separate cohorts; any inference between the two is therefore associative rather than causal. Different mapping systems were used between index and redo procedures, precluding quantitative assessment of lesion regression. These findings are limited to the 31-mm Farawave platform, and lesion characteristics may differ with other PFA systems.^30^

## Conclusion

In the acute mapping cohort, PFA produced larger antral left-sided lesions than CBA. In a separate redo cohort, chronic per-vein PV durability at approximately one year was comparable between modalities. Fluoroscopy-guided single-shot ablation may overlook incomplete or excessive lesions, highlighting the potential role of adjunctive 3D mapping for procedural optimization.

## Supplementary Material

euag167_Supplementary_Data

## Data Availability

The data that support the findings of this study are available from the corresponding author upon reasonable request.
