# Supplementary material for: Pulsed field or cryoballoon ablation for paroxysmal atrial fibrillation—insights from acute and chronic electroanatomic remapping in the randomized SINGLE-SHOT CHAMPION trial
Source: Europace. 2026 Jul 3;28(7):euag167. doi: 10.1093/europace/euag167 (PMC13385999; doi:10.1093/europace/euag167)
Supplement: euag167_Supplementary_Data [file euag167_supplementary_data.docx]

**Pulsed Field or Cryoballoon Ablation for Paroxysmal Atrial Fibrillation – insights from acute and chronic electroanatomic remapping in the randomized SINGLE-SHOT CHAMPION trial**

Thomas Kueffer* PhD^1,2^, Sven Knecht* PhD^3^, Elias Ayadi^3^, David Spreen MSc^3^, Salik ur Rehman Iqbal MD^1^, Gregor Thalmann MD^1^, Patrick Badertscher MD^3^, Jens Maurhofer MD^1^, Philipp Krisai MD^3^, Nikola Kozhuharov MD^1^, Peter Jüni MD^4^, Corinne Jufer MSc^1^, Helge Servatius MD^1^, Hildegard Tanner MD^1^, Michael Kühne MD^3^, Laurent Roten MD^1^, Tobias Reichlin* MD^1^, Christian Sticherling* MD^3^,
on behalf of the SINGLE SHOT CHAMPION investigators

MD^1^

^1^ Department of Cardiology, Inselspital, Bern University Hospital, University of Bern, Bern, Switzerland

^2^ SITEM Center for Translational Medicine and Biomedical Entrepreneurship, University of Bern, Switzerland

^3^ Department of Cardiology, University Hospital Basel, University Basel, Basel, Switzerland

^4^ Clinical Trial Service Unit and Epidemiological Studies Unit, Nuffield Department of Population Health, University of Oxford, Oxford, United Kingdom

Supplemental material:

Contents

[Supplemental Table 1: Predictors of pulmonary vein reconnection during repeat procedures after pulsed field or cryoballoon ablation. 2](#_Toc215727508)

## Supplemental Table 1: Predictors of pulmonary vein reconnection during repeat procedures after pulsed field or cryoballoon ablation.

| Pulmonary vein reconnection in patients undergoing clinically indicated repeat ablation after pulsed field ablation or cryoballoon ablation | | | | | |
| --- | --- | --- | --- | --- | --- |
| Predictor* | OR | 95% CI lower | 95% CI upper | P value |  |
| Intercept | 0.28 | 0.07 | 1.14 | 0.08 |  |
| LA size (mL) | 1.02 | 1.00 | 1.03 | 0.01 |  |
| Randomization: PFA (vs. CBA) | 0.82 | 0.40 | 1.68 | 0.59 |  |
| 3D post-ablation mapping | 0.80 | 0.38 | 1.68 | 0.55 |  |
| Ablation intensity (z-score) † | 1.42 | 1.00 | 2.04 | 0.05 |  |
| Procedure duration (min) | 0.99 | 0.98 | 1.01 | 0.22 |  |
| * Mixed-effects logistic regression with patient ID as random intercept (N = 184 veins).  † Ablation intensity was z-scored within each ablation modality (PFA, number of applications per vein; CBA, freeze duration per vein). Odds ratios correspond to a 1-SD increase. | | | | | |
